# Discourse on Exposure to Pornography Content Online Between Arab Adolescents and Parents: Qualitative Study on its Impact on Sexual Education and Behavior

**DOI:** 10.2196/11667

**Published:** 2018-10-09

**Authors:** Anat Gesser-Edelsburg, Munawar Abed Elhadi Arabia

**Affiliations:** 1 The Health and Risk Communication Research Center School of Public Health University of Haifa Haifa Israel; 2 School of Public Health University of Haifa Haifa Israel

**Keywords:** internet, pornography, porn viewing, sexuality, taboo, Israeli Arab adolescents, discourse, sexual education and behavior, sexual double standard

## Abstract

**Background:**

The internet revolution of the 21st century has made sexual content available and accessible on a scale that has never existed before. Many studies have indicated that the use of pornography was associated with more permissive sexual attitudes and tended to be linked with stronger gender-stereotypical sexual beliefs. It also seemed to be associated with other risky behaviors and sexual promiscuity. Pornography exposure in conservative societies leads to conflicts with religious and cultural taboos.

**Objective:**

The aim of this study was to characterize the barriers and difficulties that prevent sexual discourse in the Arab society and enable pornography viewing according to the perceptions of adolescents and mothers.

**Methods:**

This study involved qualitative research methods and in-depth interviews with 40 participants. This study included 20 Arab adolescents, sampled by 2 age groups (14-16 years and 16-18 years), and 20 mothers of adolescents from both sexes.

**Results:**

The findings indicate that mothers “turn a blind eye” to porn viewing and sexual activity by boys; however, they show a sweeping prohibition and denial of such behavior by girls. Boys reported viewing porn routinely, whereas girls denied doing so, but admitted that their female friends watched porn. The study also found that boys experienced guilt during and after viewing porn as a result of the clash between modernity and traditional values. The mothers and adolescents emphasized the need for an open sexual discourse to reduce violent behaviors such as Web-based sexual harassment, including sending videos and pictures of naked girls, often accompanied by threats and blackmail.

**Conclusions:**

It is necessary to find a way to encourage a significant sexual discourse to prevent the violent consequences of its absence in the Arab society. A controlled, transparent, and critical sexual discourse could help youth make more informed decisions concerning the search for sexual content, porn viewing, and sexual behavior.

## Introduction

### Sexual Discourse in the Arab Community: Between Tradition and Modernization

#### In the Arab Community, Sexual Discourse is Considered Taboo

Among Islamic religious individuals, explicit sexual discourse is not encouraged. The reason is that according to the religion and rules of the Quran, only married people are permitted to engage in sexual relations, and therefore, sexual discourse with adolescents is considered to potentially encourage premarital sex [1]. However, as Roudi-Fahimi [2] indicated in their systematic review, despite the religious prohibition, there is in fact sexual contact between young people. Therefore, awareness has grown in some of the Arab countries that the population should be given access to information about contraceptives and about sexually transmitted diseases (STDs) resulting from multiple partners [2]. However, in contrast with the recognition of the sexual activity of youth by health authorities in Arab countries, there is still a social taboo and denial by parents that Arab youth have premarital sex, and therefore, sexual discourse in the public and domestic spheres is nonexistent [3].

In reality today, Arab youth experience sexual contact, porn viewing, and premarital sex [3-6]. The main reasons are that in the last decade, Arab communities have undergone immense transformations, largely related to the infiltration of Western elements into the society, technological means that narrowed the gap between Arab and Western society, and global social and economic changes [7,8]. The process of modernization where women education increases, fertility decreases, and marital timing is postponed occurs mainly in Arab communities living in advanced democratic countries. Furthermore, Arab youth are more exposed than the older generation to the effects of the technologies that arrived with the new media revolution. Arab youth are currently living in a dual reality where on the other hand, they are attracted and yearning for the liberal technological Western culture, while at the same time, they want to maintain their allegiance to their ancestors’ traditional isolationist culture [8-10].

Studies have shown that the nature of sexual discourse, its culture, and the way it is managed have a predictive correlation with the actual behavior of adolescents and adults [11,12]. The absence of overt sexual discourse also leads to ignorance [13], fears, and anxiety in Arab adolescent boys and girls. For instance, many Arab girls reported that the appearance of signs of menstruation came to them as a complete surprise [13,14].

#### Sexual Double Standard and the Status of Women in Arab Society

The sexual double standard is the widespread belief that sexual behaviors are judged differently depending on the gender of the sexual actor [15]. The sexual double standard is policed and controlled through “the-male-in-the-head” [16]. This concept refers to male power under heterosexuality, which leads to the unequal relationship between femininity and masculinity, and relates to the control of both female and male sexuality. A characteristic of the-male-in-the-head is the silencing of sexual female voices and the simultaneous noise of male-dominated conversations in this area. Boys and men are thought to receive praise and positive attributions from others for nonmarital sexual contacts, whereas girls and women are believed to be derogated and stigmatized for similar behaviors. In other words, men are rewarded for sexual activity, whereas women are derogated for the same activity [17,18]. The sexual double standard is related to standard gender stereotypes: sex and desire are not feminine, whereas they are expected from men. Heterosexuality is constructed under a male gaze [19] so that men are in the position of power and they have access to discourses of sex and desire, whereas women’s desire is silenced. Women are supposed to hide their desire and make it invisible [19], whereas heterosexual men can express it openly. Furthermore, sex is seen as a greater risk for women because they can become pregnant, and although men can easily walk away from this situation, women have to shoulder the responsibility [18].

The sexual double standard intensifies in patriarchal societies such as the Arab society. In the Arab society, the woman is considered property of the man. Not only is her status unequal, but the desires of the man dictate her behavior. Women’s expression of sexual wishes or desires that contradict those of the man are often considered an offense against the man’s honor and the family honor [20].

Thus, men, given their superior position and their perception of women as their property, often rape women. It is important to emphasize that the very concept of rape does not exist in many Arab countries and that the act that is punishable is extramarital sex (a man is definitely allowed to rape his wife) [21,22]. According to the laws in these countries, 4 witnesses are often required to make a charge of rape. In the absence of 4 witnesses, the most important evidence to support a charge of rape or, alternately, extramarital sex is the woman’s pregnancy as a result of the rape. The woman is accused and punished for being raped, whereas the man is not accused at all. The situation clearly discriminates against women and places a double punishment on the victim [23]. In some Arab countries, to “ease” the woman’s plight, she is not punished but rather ordered to raise the child without a father (of course abortion is not permitted, even in cases of rape) [24]. Another issue associated with double standards toward women is murder on the background of so-called family honor, which is also familiar in the Arab society in Israel. Although men are not judged for having sex before or outside of marriage, women are judged by the society and even murdered for what is defined as “improper sexual behavior” [25].

### The Internet as a Source of Sexual Information and Consumption

Internet-enabled devices have enabled people of all ages to consume sexual information at an availability and speed that have impacted and changed the sexual habits and knowledge of adolescents [26,27].

The internet is considered a more highly sexualized environment than other media [28], and research has shown that the number of youth who intentionally or accidentally encounter pornographic material online has risen significantly [29,30].

The internet occupies a prominent and prioritized place in the lives of many youth [29,31,32]. For example, a survey by the World Internet Report of people aged 12 to 14 years from 13 different countries found that 100% of British, 98% of Israeli, 96% of Czech, and 95% of Canadian youth reported using the internet regularly [33].

The internet can serve youth as a source for the acquisition of skills, the development of high literacy, and entertainment [34]. However, at the same time, for users with certain sociodemographic characteristics, it can be a source of risk behaviors, such as pornography viewing and addiction [35,36].

### Pornography and Youth

The legal status of pornography in the world varies widely from one country to another [37], but attempts to restrict access to online pornographic contents in different countries have usually failed due to the ease of access [38]. Systematic studies and reviews have indicated that youth view porn from 10 years to 18 years, although prevalence rates between studies varied greatly [39].

Young adults can also be exposed to pornographic contents “by chance” when they do not intend to [40-42]. Exposure to sexually explicit material in adolescence has a unique impact because in adolescence, youth feel high uncertainty about their identity and sexual boundaries [43]. Furthermore, exposure to porn from a young age impacts the way youth think about sexuality as well as their actual sexual behavior. According to a large survey of American college students, 51% of males and 32% of females admitted to viewing pornography for the first time before they were 13 years old [44]. For teenagers exposed to pornography within a family setting, pornography causes stress and increases the risk for developing negative attitudes about the nature and purpose of human sexuality.

For adolescents who view pornography, their attitudes toward their own and others’ sexuality change, and their sexual expectations and behavior are shaped accordingly [43-45]. A study of 2343 adolescents found that sexually explicit internet material significantly increased their uncertainties about sexuality [43].

Age groups of 14-16 years and 16-18 years are sensitive ages for porn viewing because from age 14 years, adolescents face growing social pressure from their peer group to have romantic partners [46,47]. Relations with partners at these ages are influenced by what they viewed and learned from porn.

Due to the pervasiveness of online porn in the cultural and social environment of adolescents, a systematic survey was held in 2016 [48], which found (despite the differences in the methodologies of the different studies) that pornography use was associated with more permissive sexual attitudes and tended to be linked with stronger gender-stereotypical sexual beliefs. It also appeared to be related to the occurrence of sexual intercourse, greater experience with casual sexual behavior, and more sexual aggression, both in terms of perpetration and victimization.

Porn viewing often leads to adolescents’ lower levels of sexual self-esteem [49], more liberal sexual positions, and a higher belief that peers are sexually active, raising the likelihood of younger sexual initiation [26].

Adolescents who are exposed to sexual behaviors outside of cultural norms may develop a distorted perception of sex as unrelated to love and intimacy and a desire for sexual engagement without emotional commitment [50]. The combination of peer pressure, porn viewing, and patriarchal values leads to risky behavior [51].

Studies have indicated that mostly boys but also girls tend to engage in more “sexting” (exchanging sexual text messages) when they view porn. Sexting by youth often leads to sexual contempt and online sexual violence. Studies indicate that when sexting is accompanied by alcohol drinking, it leads youth to a loss of control and potential sexual violence [52,53]. Moreover, adolescents who are exposed to pornography may develop positions that support the “rape myth,” which ascribes responsibility for sexual assault to the female victim [26,54].

There are few studies on the viewing habits of sexual contents and use of pornography in Arab countries among young adults in general and adolescents in particular. Studies that have examined the subject have found that the internet exposes Arab youth to contents that contradict religious and cultural taboos. The studies found that because of the proscription and supervision in Arab countries, youth acquire information and view pornography clandestinely [55].

Arab youth living in conservative communities watch porn secretly not only out of the psychological fear of the reactions of their parents and other authority figures in their lives [43] but also because of the religious proscription that does not exist for secular youth living in liberal societies [56].

It was found that because Arab adolescents live in a conservative world with a culture of silencing, their level of emotional readiness and tools to filter sexual contents are much lower than that of Western youth [13,57]. For instance, in a study of young adult students in Lebanon, it was found that a considerable number of them used the internet to view pornography and gamble [58].

Many studies are conducted around the world on pornography consumption. However, as stated in the systematic survey by Owens et al [59], it is necessary to continue studying this phenomenon by encouraging global studies. Few studies have been conducted on Arab youth concerning sexual discourse on the social networks and the use of technological means. Most of the studies in the literature are quantitative studies that indicate the frequency of porn viewing and/or the attitudes and perceptions of youth about specific issues on which they were asked in closed questionnaires. There are very few qualitative research studies, namely, in-depth “face-to-face” interviews that take a deep look at the features of the sexual discourse between Arab youth and their parents as well as the gaps and conflicts that arise from them.

In light of the sparsity of data specifically concerning this population, this study will contribute to understanding the impact of social, cultural, and religious patterns on the perceptions of online sexual discourse of Arab youth and mothers in Israel. Moreover, the study can provide a basis for the formulation of recommendations that put an emphasis on risk communication on the internet with the purpose of promoting a policy of effective and accommodated sexual discourse for the needs of Arab youth and their parents.

### Objectives

The objective of this study was to characterize the barriers and difficulties that prevent sexual discourse in the Arab society and enable pornography viewing according to the perceptions of adolescents and mothers.

## Methods

### Study Design and Analysis

This study employs qualitative research, which allows the in-depth observation of a phenomenon through the prism of the study participants. The purpose of the phenomenological qualitative research method is to understand the studied phenomenon by analyzing the experiences of a certain population, with an emphasis on selecting an informative group that authentically represents it [60].

### Research Population

A total of 40 respondents were interviewed for this study. This study comprised 20 Arab adolescents (Table 1) in 2 age groups, who, according to the literature, are in different developmental stages: 14-16 years and 16-18 years [61]. In addition, 20 mothers (Table 2) of adolescents of both sexes were interviewed. Only mothers were chosen and not fathers on the assumption that men in the Arab society would refuse to have a conversation on sexuality in general, and especially the fathers of daughters.

### Recruitment and Interview Process

An application was submitted to the Faculty of Social Welfare and Health Sciences Ethics Committee for research with human subjects at Haifa University, and full ethical approval (no.439/17) was granted. Participants were recruited through purpose sampling of Arab schools in Nazareth, Kafr Sullam, Reina, Kafr Nin, and Ein Mahel. These schools were chosen to achieve a heterogenic profile of the youth population. The researchers approached different schools in Nazareth and its surroundings to sample youth from different ethnicities—Muslims and Christians. It is important to note that the youth in Nazareth live in a mixed urban environment including Jews. This environment is fundamentally different from the isolation of the purely Arab population such as in the villages of Kafr Sullam, Reina, Kafr Nin, and Ein Mahel.

The mothers of boys and girls were approached through class WhatsApp groups. The approach laid out the research goal and provided the contact information of 1 of the researchers and an invitation to contact her. The researcher asked the mothers for permission to interview their children. Following the mothers’ approval, the researcher contacted the adolescents and asked for their consent to participate in the study. In addition, mothers were approached separately. It should be noted that it was decided not to interview the adolescents whose mothers agreed to be interviewed to allow interviewees to speak freely. The interviews with the adolescents were conducted wherever the interviewees felt comfortable, usually in their homes or in parks.

**Table 1:** Adolescent interviewees: sociodemographic data.

Sociodemographic characteristics		Statistics
**Gender, n (%)**		
	Men	11 (55)
	Female	9 (45)
Age (years), mean (SD); range		16.1 (1.1); 14.0-18.0
**School level (grade), n (%)**		
	8	2 (10)
	9	3 (15)
	10	2 (10)
	11	10 (50)
	12	2 (15)
**Religion, n (%)**		
	Muslim	19 (95)
	Christian	1 (5)
**Place of residence, n (%)**		
	Nazareth	15 (75)
	Kafr Sullam	3 (15)
	Reina	1 (5)
	Kafr Nin	1 (5)

**Table 2:** Sociodemographic data of mother interviewees^a^.

Sociodemographic characteristics		Statistics
Age (years), mean (SD); range		41.1 (3.7); 35.0-47.0
Education (years), mean (SD); range		13.6 (2.3), 11.0-20.0
**Employment, n (%)**		
	Income tax	2 (10)
	Teacher	3 (15)
	Housewife	8 (40)
	Nurse	2 (10)
	Cashier	2 (10)
	Clerk	1 (5)
	Secretary	1 (5)
	Doctor	1 (5)
**Place of residence, n (%)**		
	Ein Mahel	1 (5)
	Nazareth	17 (85)
	Daburiyya	1 (5)
	Yafia	1 (5)
**Level of religiousness, n (%)**		
	Secular	3 (15)
	Traditional	10 (50)
	Religious	7 (35)
**Income level, n (%)**		
	Low	3 (15)
	Medium	12 (60)
	High	5 (25)
Number of children, mean (SD); range		3.6 (1.6); 1-6

^a^All mother interviewees were born in Israel and they are Muslim by religion.

The interviews with the mothers were conducted in their homes. The interviews lasted between 45 min and 1 hour and were conducted by 1 of the researchers who was trained to conduct qualitative interviews. The interviews were recorded and transcribed.

### Research Tools

The choice of personal interviews rather than focus groups was made to give the interviewees the confidence to speak freely about a sensitive subject. Semistructured protocols were prepared for the interviews, adjusted to the research subpopulations. The interviews were held in Arabic, the participants’ mother tongue. Moreover, 2 protocols were designed for this study: for the adolescents and the mothers. The protocols for the Arab adolescents included questions on the perception of sexual discourse with peers and parents, information searching about sex and sexuality, and pornography viewing. The protocols for the interviews with the mothers included questions about their relationships with their adolescent children, sexual discourse at home, sources of information about their children’s sexuality, and sex education.

### Data Analysis

The findings were analyzed by the content analysis approach [62] using the following process: in the first stage, themes were analyzed and coded for each population, adolescents and mothers, separately, while identifying main themes and subthemes. In the second stage, themes that arose among the 3 research groups—adolescents aged 14-16 years, adolescents aged 16-18 years, and mothers—were analyzed and coded. In the third stage, each subgroup was integrated separately; all of the interviews of the adolescents in each age group and of the mothers were integrated separately. In the final stage, integrated super-categories were constructed for all of the research populations.

### Validity and Reliability

Interviews were recorded, transcribed, and logged in a field diary. This enabled examination of the reliability of the data received from the participants and control of the analysis of the findings by the researchers [63].

The field diary included notes of the time and the place of the interview, the dynamics during the meeting, resistance by interviewees to questions in the interview, and nonverbal reactions (such as body gestures or facial expressions) that cannot be surmised from the transcript of the interview. Given the sensitivity of the subject of sexuality both for the youth and the mothers, the researchers’ documentation and reflection on the process was a tool for correcting and improving the discourse with the interviewees as well as providing a holistic and deeper picture of the data.

The interview protocols were designed in Hebrew and translated into Arabic, the mother tongue of the research population, and then translated back from Arabic into Hebrew to check the wording. The interviews were transcribed in Arabic by 1 of the researchers who is fluent in both Arabic and Hebrew. Likewise, a number of stages of data collection and analysis were performed: a pilot to test protocols with 2 mothers and 2 adolescents, joint meetings of the researchers during the data collection process, reading of transcripts by the 2 researchers separately, and decision of categories and subthemes through an agreement between the researchers. Moreover, study participants represented different subpopulations (adolescents by age groups and mothers), which can strengthen the credibility and validity of the findings in relation to the studied phenomenon [62].

## Results

### Main Findings

The main findings that arose from the interviews with the youth and the mothers indicate 4 central themes. The first theme is the absence of sexual discourse between adolescents and their parents. The technological internet revolution has led to the availability and accessibility of sexual contents but did not advance the discourse between youth and their parents, and sexual discourse is still a social taboo. The second theme included barriers that prevent sexual discourse: normative, religious, cultural, and psychological (see details below). The third theme is that the internet realm presents a unique conflict for Arab youth from a conservative society between attraction to pornography and traditional norms. The fourth theme is the consequences of porn viewing—sexual aggression.

### Absence of Sexual Discourse Between Adolescents and Their Parents

All the adolescents (n=20), without exception, stressed that sex and sexuality are taboo and there is no sexual discourse between them and their parents. For instance, 1 of the boys said:

In our society parents do not talk about sex. They perceive the subject as sensitive and forbidden, and therefore we as teenagers look for another way to understand the world of sex...

Similarly, Arab mothers (n=20) also stressed the fact that the subject of sexuality and sexual discourse is a social taboo and that is one of the reasons for the absence of sexual discourse with their children. For instance, 1 of the mothers said:

I don’t know any parents who have a sexual discourse with their teenaged children. In our society it is forbidden to talk about it. You leave it until they get married and then they learn everything by themselves... Our society does not talk about such things.

Most of the mothers in the study (n=18) had instrumental sexual discourse with their daughters, which was limited to physiological development, but they did not discuss physiological changes with their sons. One of the mothers said she explains the physiological changes to her daughters and lets her husband talk to their sons:

Yes, we do discuss issues related to adolescence, the changes that occur in your body, I discuss “periods” with my daughters more than with my sons. I don’t talk to them, it’s hard for me! When it comes to boys, I leave it to their father, even though most of the time he does not show any interest.

The interviews in the study found that some mothers (n=14) emphasized that the conversation with the boys focused only on STDs to warn and scare them about the consequences of having sex “before marriage.” For example, 1 of the mothers of sons said:

The most important thing for me is to talk about STDs like AIDS. I keep on scaring him that it is an incurable disease. Whoever gets AIDS has a slow death, is rejected by our society. A person who has that disease is perceived as disgusting, perverted, and having had “forbidden” sex. I use the intimidation mechanism to make sure he doesn't have sex.

Table 3 presents the barriers raised by interviewees concerning the absence of sexual discourse with their children.

### Conflict: Attraction to Pornography Against Traditional Norms

The adolescents said that curiosity and the absence of discourse at home led most of them to seek information on the internet and especially to watch porn. All the boys in the interviews (n=10) reported that they watch pornographic movies. For example, 1 of the boys said:

My friends at school go into those sites... Porn sites. They watch everything that has to do with sex. Intercourse and so on. Because they want to get to know that world.

As for girls, a more complex picture arose from the interviews. On one hand, most of the girls (n=6) denied viewing porn, but on the other hand, all the girls stated that their female friends did. It can be assumed that not all the girls indeed watch porn, but because of embarrassment to admit to it directly, they prefer to state that their female friends do it. In addition, the girls state their attraction and repulsion over dealing with sexuality.

**Table 3:** Barriers that prevent sexual discourse.

Barrier and subheadings		Selected response
**The normative barrier**		
	Conducting sexual discourse is forbidden	“Our society is very conservative. They see this kind of subject as something wrong.”
	Issues and dilemmas could not be raised in the public domain and social networks	“I don’t know, if the media talks about such subjects I think it is done very superficially because otherwise they would get unbearable criticism.”
**The religious barrier**		
	Sex is “forbidden in Islam”	“No, of course not. It is a very sensitive subject. Thank God me and my daughters are religious. We don’t think about sex or sexuality.”
	Effect of religion on the absence of discourse	“Religion is also a main factor. The Islamic religion does not allow us and our parents to talk about sex. We know that it is wrong to view those movies because even viewing is adultery according to Islam. It is called ‘visual adultery’ but we still do it.”
**The cultural barrier**		
	Absence of discourse is passed through generation	“Because we got used to it. Our parents did not talk to me about anything to do with sex. So I don't talk either.”
	Awareness among adolescents of the history of absence	“Our parents don’t talk, they are afraid of such subjects, because they got used to it. Their parents didn’t talk either. So they feel that such a conversation is wrong.”
**Discourse as a psychological barrier**		
	Shyness and unacceptability of sexual discourse	“Sexual discourse is not acceptable in our society like other subjects. It comes from the culture of shyness we grew up in. We are the parents, mothers and fathers, so how could we transmit or talk about such a sensitive subject with our children if we don’t have the right education ourselves? We learned about this from our personal experience.”
	Embarrassment over touching sexual subjects among adolescents	“There are lots of boys who are embarrassed to ask their parents and there are some who are afraid of punishment.”
**Giving legitimacy to sexual activity**		
	Concerns and fears of encouraging unacceptable sexual behaviors	“No, I did not talk about safe sex, like using a condom, because that would encourage them. As a result, I prefer not to talk about safe sex. If I am talking about a condom then he won’t listen to me when I talk about sex being forbidden and STDs^a^. If I talk about a condom then he will rely on it and won’t mind having sex.”
**The age barrier**		
	Children aren’t mature enough to talk with them about sex (age group 14-16)	“No, my husband and I do not talk about sex. I think there is time for that. But I saw my son watch sexual movies! Yes, I punished him. Because he is still too young for such things. They’re pretty young, 9, 14. I think it doesn’t make sense to talk about sex. I’m sure they can’t process it. Why confuse them?”
**Fear of their husbands’ reactions to having sexual discourse at home**		
	Said sexual conversations at home are not at all acceptable to husbands	“No way, of course not!! My husband would kill me if he heard such a thing at home. He would start shouting right away, breaking everything he saw, that's why I prefer not to talk about such things. We got used to not talking with the children about things like love and sexuality.”

^a^STDs: sexually transmitted diseases.

For example, 1 interviewee said:

I always thought that pregnancy occurred the moment a man and a woman kiss. Or when a woman drinks water from the man’s glass. They explained to me that my information was wrong. They told me the truth. I didn't like the conversation and as a result I left the conversation/the group.

The interviews indicate that most of the adolescents expressed an inner conflict between their attraction to viewing porn and traditional values. Most of the boys (n=9) reported feeling guilty because of the conservative education they received from their society and parents. For example, 1 of the boys stressed:

On the one hand we know it is forbidden, on the other hand we want it and need it. And you feel guilty every time you watch.

Similarly, another boy shared that:

There is an internal conflict and problem of conscience, because on the one hand the boys want to watch movies and know everything, to experience the experience and feelings, and on the other hand they know it is wrong and forbidden by the religion, our parents don’t accept it.

There were boys (n=7) who reported that they do not feel guilty while watching porn, but they do feel guilty only after they finish watching it:

While watching there is no conflict because we are focused on the movie. The inner conflict, the guilt, between knowing that it is forbidden and consuming porn, appears after the movie is over.

As aforesaid, the girls said that their friends watch but they do not. They mentioned the guilt that goes with watching porn. One interviewee said:

I think they feel guilty, because they know this is all against our culture and values. I’m sure the conflict is much worse for the girls, because our society puts an emphasis and is afraid of anything that happens to a girl. You know and probably heard about cases of girls being murdered, therefore girls do it secretly and experience greater conflict.

The interviews with the mothers indicated that the boys’ mothers know that they watch porn, whereas the girls’ mothers tended to deny that their girls did so. One mother said there is a difference between what the patriarchal Arab society allows boys and girls:

We as mothers are aware that our boys watch porn and talk about what they watched with each other, but we ignore it and move on! But in Arab society that is not the case for girls. We impose all of the housework on them, in addition to schoolwork, so that they don’t have time to think about ‘sexual desire’. Some prefer to marry them off young to maintain family honor.

It emerged among the adolescents that despite risky behaviors such as drinking alcohol and viewing porn, premarital sex is still a significant barrier for them. Boys (n=9) noted that they oppose premarital sex because it disrupts the correct order of relations:

Of course, I’m against premarital sex because if we do it before marriage, the desire for marriage goes down, and in the end most of the youth will not get married.

Some of the youth (n=18) and the mothers (n=20) explained that they opposed premarital sex because of the Islamic religion, which proscribes sexual relations without religious sanction before marriage. One mother said:

I’m against premarital sex. First of all it’s forbidden by our religion. Secondly it’s unacceptable in our society. Thirdly I think it violates the trust between the girl and her parents.

Family honor is also 1 of the main barriers that prevent youth from having premarital sex. One of the boys described it as follows:

Our society does not accept it. It is “merciless” and if they find someone who had sex the result is “suicide” or banishing from a certain area.

Moreover, 1 of the boys said that if a girl had premarital sex she would be portrayed as “used goods”:

Men are allowed to do everything, even premarital sex. On the other hand, girls are not allowed to have premarital sex because otherwise they are perceived as secondhand.

Likewise, girls said that if a girl becomes pregnant before marriage, she has no future. For instance:

Our parents taught us that a girl who has sex before marriage will never get married. Because no man will accept that.

As for getting pregnant before marriage, all the mothers, especially mothers of girls (n=17), emphasized the sensitivity of the subject and said such an event could have a heavy price.

The adolescents emphasized that a girl who gets pregnant before marriage does not go to her parents to find a solution to her problem. Some of the boys (n=8) declared that the girl would ask for help from her boyfriend. For instance:

I think she would go to whoever she had sex with and they would think together about how to get an abortion. If the boy denies or evades then I think she would go either to her girlfriend or sister. Or she would hide and conceal the pregnancy and abort it without anyone knowing.

Other adolescents, especially girls (n=9), thought the girl would not go to anyone for help because nobody could help her. For instance:

That’s a very difficult situation. I don’t know if she would talk, nobody could help her, I think she would find a solution by herself.

However, some of the girls emphasized that despite fear of their parents, they would be the only people who could help the girl:

How hard it is also depends on her age. If she were 18 it would be less complicated than if she were 16 or 17. I think she would go to her parents because in such a situation only her parents would be able to help.

### The Consequences of Porn Viewing—Blackmail and Sexual Abuse

Although the mothers of boys turned a blind eye, most of the mothers (n=16) expressed fears and concerns about the movies their sons watched and their consequences for their children’s sex education:

You need to understand that life isn’t like a movie. Both the sex and the way they have sex are presented in a really disgusting way, and as a result they look at sex completely differently than in life. I don’t think the movies they watch have fair information. Viewing causes addiction and divorce. I know about a lot of cases when a husband and wife broke up because he asked her to do things like what he watched. This will cause conflict and end with divorce.

According to adolescents, unchecked exposure to pornographic movies and sexual contents also leads to blackmail, sexual harassment, and sexual abuse. For instance, most of the adolescents (n=18) mentioned online sexual harassment by sending videos and pictures of naked girls. One of the boys said:

Sexual abuse is not only rape, today there are many cases of boys and girls threatening and blackmailing each other, such as with pornographic pictures and films. Today there is a phenomenon of girls sending pictures of themselves naked.

One mother discussed how the absence of sexual discourse leads to sexual exploitation and abuse in the Arab society:

Frequently we do not allow the girls to know about sex, and on the other hand we are aware that all of the boys watch and look for sexual information online. I’m talking about pornographic sites. Most of the information they get is wrong. It is reflected by the rate of sexual harassment and cases of rape, which we hear about every day on the news.

Most of the mothers of girls (n=16) emphasized the vital importance of warning their daughters against online sexual abuse because of the availability and accessibility of apps that help rapidly distribute all of those illicit pictures and videos:

I told her we are not allowed to take pictures of ourselves and send to WhatsApp groups because there are a lot of people who take advantage of those pictures and change them.

Boys (n=9) and girls (n=7) stressed that the absence of sex education at school leads them to look for information from other sources and that sex education in the framework of the school could potentially help youths:

It is very important to talk in school because our society does not talk and does not let us talk about sex or sex education. There is no awareness of these sensitive issues. We as teenagers go and look in the wrong places. With sex education you could raise a whole generation with a better outlook on sex.

Another advantage of sex education that was mentioned by a considerable number of boys (n=10) has to do with reducing sexual abuse and other risky sexual behaviors. For instance:

I think it is very important because we as boys and girls don't know where to get this information. Our parents don’t talk and they don’t talk in school either. There needs to be at least one source to aim us in the right direction. And there is a good chance that lectures about sex education would reduce the cases of sexual abuse, rape and so on.

## Discussion

### Principal Findings

In spite of many changes occurring in Arab societies as a result of their connection with the West, the subject of sexuality is still a taboo [64]. The taboo was reflected in this study as well. The adolescents and the mothers in this study mentioned the religious, cultural, and psychological barriers that make it difficult for them to discuss sexuality in the family setting. The discourse of sexuality in adolescence is limited only to certain physiological aspects such as girls getting their periods. The main perception is that sexuality must not be discussed, that premarital sexual contact is forbidden by religion, and that sexual discourse can legitimize premarital sex. The literature indicates that despite the religious and cultural proscriptions, Arab youth do have premarital sex [4,64]. The interviews with adolescents and mothers in this study also found that the actual reality is different from the conservative perception. On the surface, the mothers note that the proscription of sexual activity by adolescents is proscripted, but mothers’ warnings to their boys to be careful of sexual relations because of the fear of contracting STDs indicate that they take into account that adolescent boys will have premarital sex. The findings of this study strongly indicate the prevailing patriarchal worldview [22,65].

The findings of this study indicate the phenomenon of “sexual double standard,” meaning the widespread belief that sexual behaviors are judged differently depending on the gender of the sexual actor [15]. Boys and men are thought to receive praise and positive attributions from others for nonmarital sexual contacts, whereas girls and women are believed to be derogated and stigmatized for similar behaviors. In other words, men are rewarded for sexual activity, whereas women are derogated for the same activity [17,18]. Similarly, in this study, boys and girls said that the girl is the one who would pay the highest price. It is the girl who would be condemned familially and socially; moreover, her life could be in danger, as a result of compromising family honor. The findings presented in this study are compatible with the research literature that shows that men in the Arab society have much more sexual freedom to act without harming family relations, as opposed to women who must subdue to many dictates to maintain domestic peace and family honor [65]. It is important to note that the patriarchy of the Arab society is also reflected by its reported online pornography consumption [66]. The boys in this study reported that they viewed porn, as opposed to the girls, who denied doing so but admitted to doing so indirectly by reporting that their female friends did.

These findings indicate the internalization of the gender stereotype by the youth, that is, sex and desire are not feminine; however, they are expected from men. Heterosexuality is constructed under a male gaze [19]. Thus, men are in the position of power and they have access to discourses of sex and desire, whereas women’s desire is silenced. In addition, the findings of the study indicate the internalization of the sexual double standard by the mothers in this research. As Milhausen and Herold [15] point out, men are not the only ones who internalize double standards—in many cases, women do so as well.

Mothers in this study tended to ignore the fact that their sons viewed porn; however, they denied that their daughters might behave the same way. Supposedly, there is a sweeping prohibition against sexual contact and porn watching both for boys and girls, but mothers’ lenient attitude toward the behavior of male adolescents emphasizes the prevailing objectification of adolescent girls. It is precisely the mothers, female adults, who are the ones who internalize the patriarchal view. They maintain that it is mainly women who are under the imperative to avoid becoming “bad” girls who have sexual desire and engage in sex with whomever they please [19]. They maintain that women should be judged more harshly than men for sexual activity and that women should “respect” themselves more [67].

Moreover, some of the mothers in this study reported that they avoid talking with their children because they are afraid of the wrath of the father in the family who would not tolerate such conversation. In addition, what arises from this study is the overt and covert discourse that interacts with other risky behaviors in the Arab society, namely, the sweeping prohibition versus what actually happens. For example, the prohibition in Islam on drinking alcohol versus the secret drinking by Muslim youths while parents turn a blind eye [68].

The findings of this study also indicate the ambivalence and internal conflict youth feel about viewing porn. Adolescents feel guilty during and after viewing. They say these feelings arise because of the moral conflict between modernity and traditional values. The fierce internal conflict they feel corresponds with the studies that indicate the duality in which Arab adolescents experience the clash between modernization and traditional values [10]. This clash is reinforced by the new media revolution, which made sexually explicit contents accessible in a way that no other media had ever done before. Furthermore, viewing porn influences the way youth discuss sex among themselves and the way they actually behave. Adolescents reported sexual abuse that occurs in their social realm following porn viewing. Unchecked exposure to pornographic movies and sexual contents leads, according to the interviewed adolescents, also to blackmail, sexual harassment, and sexual abuse. These behaviors have also been found in other studies of youth throughout the world [12,59,69] and in the Arab society in particular.

### Limitations

The limitations of this study are that it is a qualitative study, and therefore, it cannot represent the whole population. However, only qualitative research could make it possible to conduct an in-depth conversation about sexuality, an issue which is a social taboo. Given the extreme sensitivity of the subject, interviews could not be held with fathers.

Follow-up studies might also manage to include interviews with fathers to shed light on the issues of sexual discourse and porn viewing. It is very important to try to conduct follow-up studies on the way the sexual discourse is conducted and how it influences sexual behavior and domestic violence. Follow-up studies might design a quantitative measure assessing risky behaviors in the different teen groups.

### Conclusions

It is evident in light of the studies that this struggle between the conservative and modern cultures, which plays out within the psyches of the adolescents; the absence of sex education; adolescents’ need to search for information; and their unchecked exposure to online porn all highlight the need to change the discourse and provide effective tools to deal with this conflictual situation. The conclusion and recommendation that arises from the study is that it is not enough to transmit information and factual data as has been done so far by the school system. It is necessary to find a way to encourage a meaningful conversation to prevent the violent consequences of its absence. Introducing a sexual discourse and managing it in a controlled, transparent, and critical manner could help youth make more informed decisions concerning the search for sexual contents, porn viewing, and sexual behavior.

## Abbreviations

STD: sexually transmitted disease
